# Validation and psychometric properties of the commitment to hip protectors (C-HiP) index in long-term care providers of British Columbia, Canada: a cross-sectional survey

**DOI:** 10.1186/s12877-017-0493-5

**Published:** 2017-05-03

**Authors:** Alexandra M.B. Korall, Judith Godin, Fabio Feldman, Ian D. Cameron, Pet-Ming Leung, Joanie Sims-Gould, Stephen N. Robinovitch

**Affiliations:** 1 Injury Prevention and Mobility Laboratory (IPML), Simon Fraser University, 8888 University Drive, Burnaby, BC V5A 1S6 Canada; 2 Centre for Hip Health and Mobility, 7th Floor, 2635 Laurel Street, Vancouver, V5Z 1M9 BC Canada; 3 Geriatric Medicine Research Unit, Nova Scotia Health Authority, 5955 Veteran’s Memorial Lane, Halifax, NS B3H 2E1 Canada; 4 Patient Safety and Injury Prevention, Fraser Health Authority, Suite 400, 13450 102nd Avenue, Surry, BC V3T 5X3 Canada; 5 John Walsh Centre for Rehabilitation Research, Kolling Institute of Medical Research, University of Sydney, St Leonards, NSW 2065 Australia; 6 New Vista Care Home, 7550 Rosewood Street, Burnaby, BC V5E 3Z3 Canada; 7 Department of Family Practice, University of British Columbia, 5950 University Boulevard, Vancouver, BC V6T 1Z3 Canada

**Keywords:** Hip fracture, Hip protector, Adherence, Commitment, Long-term care

## Abstract

**Background:**

If worn during a fall, hip protectors substantially reduce risk for hip fracture. However, a major barrier to their clinical efficacy is poor user adherence. In long-term care, adherence likely depends on how committed care providers are to hip protectors, but empirical evidence is lacking due to the absence of a psychometrically valid assessment tool.

**Methods:**

We conducted a cross-sectional survey in a convenience sample of 529 paid care providers. We developed the 15-item C-HiP Index to measure commitment, comprised of three subscales: affective, cognitive and behavioural. Responses were subjected to hierarchical factor analysis and internal consistency testing. Eleven experts rated the relevance and clarity of items on 4-point Likert scales. We performed simple linear regression to determine whether C-HiP Index scores were positively related to the question, “Do you think of yourself as a champion of hip protectors”, rated on a 5-point Likert scale. We examined whether the C-HiP Index could differentiate respondents: (i) who were aware of a protected fall causing hip fracture from those who were unaware; (ii) who agreed in the existence of a champion of hip protectors within their home from those who didn’t.

**Results:**

Hierarchical factor analysis yielded two lower-order factors and a single higher-order factor, representing the overarching concept of commitment to hip protectors. Items from affective and cognitive subscales loaded highest on the first lower-order factor, while items from the behavioural subscale loaded highest on the second. We eliminated one item due to low factor matrix coefficients, and poor expert evaluation. The C-HiP Index had a Cronbach’s alpha of 0.96. A one-unit increase in championing was associated with a 5.2-point (*p* < 0.01) increase in C-HiP Index score. Median C-HiP Index scores were 4.3-points lower (*p* < 0.01) among respondents aware of a protected fall causing hip fracture, and 7.0-points higher (*p* < 0.01) among respondents who agreed in the existence of a champion of hip protectors within their home.

**Conclusions:**

We offer evidence of the psychometric properties of the C-HiP Index. The development of a valid and reliable assessment tool is crucial to understanding the factors that govern adherence to hip protectors in long-term care.

## Background

Falls persist as the leading cause of injury-related hospitalizations and deaths among individuals aged 65 years and older [1–4]. Second only to traumatic brain injuries, hip fractures are the most debilitating injury caused by falls, associated with morbidity, compromised quality of life, fear, delirium, depression and even death [5–11]. Older people residing in long-term care (LTC) are generally frail [12, 13] and are up to 10-times more likely to suffer a hip fracture during a fall than community dwelling seniors [14–16]. In the event of a hip fracture, 1 in 3 residents in LTC will die by six months, after which time, about a third of survivors will lose the ability to walk independently [7]. In Canada, the direct cost to treat a single hip fracture is estimated at $40,000, and as approximately 28,000 Canadian elders are hospitalized for hip fractures each year, the collective financial burden of hip fractures in terms of direct costs exceeds $1.1 billion annually [3, 17].

Hip fractures arise from a combination of intrinsic, situational, and environmental factors. Although the strength and integrity of bone play an important role in determining risk for hip fracture, the strongest single predictor of hip fracture is a sideways landed fall, with risk increasing 32-fold if direct impact occurs to the lateral aspect of the pelvis [18–21]. According to analysis of real life video footage of 520 falls in LTC by 160 residents, hip impact occurs in about 40% of falls, usually onto hard flooring [22].

Consisting of soft padding or hard shield domes embedded in garments or undergarments, hip protectors represent a non-pharmaceutical, and potentially cost-effective approach for hip fracture prevention (e.g., [23–27]). Rather than preventing the fall itself, which has proved challenging in LTC [28], the rationale behind hip protectors is to minimize the risk of hip fracture associated with falling, by absorbing and diverting impact energy away from the proximal femur during a sideways landing [29, 30]. If worn at the time of a fall, certain models of hip protectors have been found in clinical trials to reduce the risk of hip fracture between 69 and 80% [31–33]. When assessed solely on an intention-to-treat basis, however, the clinical value of hip protectors is compromised by rather poor adherence in the wearing of these devices, ranging from 20% (e.g., [34]) to 80% (e.g., [35]), and often below 50% in clinical trials [36]. Thus, poor user adherence in wearing hip protectors is a major barrier to their effectiveness.

Determinants of adherence with hip protectors span different socio-ecological levels [37]. Because the majority of residents in LTC have some form of cognitive impairment (e.g., [38]) and all require at least partial assistance performing activities of daily living, the attitudes and subsequent behaviour of care providers is believed to be important in determining whether a resident will wear hip protectors on a regular basis (e.g., [39–46]). For example, care providers are responsible for identifying residents likely to benefit and tolerate hip protectors, educating residents and family about the benefits of hip protectors, monitoring for signs of discomfort and pain, implementing interventions to optimize adherence, and continuously reassessing for eligibility [47]. In a recent systematic review on factors affecting use of hip protectors among residents in LTC, the commitment of care providers to hip protectors emerged as a facilitator of acceptance and/or adherence in nearly half of studies (46%) [37]. However, our understanding of the nature of commitment, along with its associated antecedents and outcomes, is constrained by the absence of a psychometrically valid assessment tool, and subsequently, reliance on relatively low-level evidence, namely expert opinion. Therefore, our aim is to develop a valid and reliable tool to measure commitment to hip protectors among paid care providers in LTC.

### Theoretical framework

Over the past few decades, researchers have conceptualized commitment to many different workplace foci, including work organizations (e.g., [48, 49]), work teams and leaders (e.g., [50, 51]), occupations and professions (e.g., [52]), organizational change (e.g., [53]) and technological change (e.g., [54]). And yet, there remains considerable uncertainty surrounding how to define and measure commitment in the workplace, how commitment in the workplace develops, and how commitment subsequently affects organizational behaviour [55]. However, what is largely undisputed is that commitment to any workplace foci should be conceived as a strictly attitudinal phenomenon (e.g., [49, 56, 57]).

According to prevailing theoretical frameworks in social psychology (e.g., ABC model), attitudes have three different components: an affective component reflecting an individual’s feelings and emotions about a target, a cognitive component reflecting an individual’s knowledge and beliefs about a target, and a behavioural component reflecting an individual’s readiness to act or behave in a certain way (e.g., [58, 59]). Thus, it follows that commitment is also reflected by a combination of affective, cognitive, and behavioural components, whereby: affective commitment refers to an emotional attachment to and identification with one or more targets; cognitive commitment refers to an internalization of the targets’ goals, norms, and values; behavioural commitment refers to a generalized behavioural pledge to serve and enhance the targets’ interests [57]. Summarized nicely by Solinger, van Olffen and Roe [57], “…thus, commitment does not come cheap: it is a binding vow, a generalized behavioural pledge to act in the interest of the [target]” (pg. 80).

Consistent with extant research conceptualizing commitment in the workplace as a purely attitudinal phenomena, we defined commitment to hip protectors as a care provider’s attachment to and behavioural intentions towards hip protectors, reflected by three components: (i) a belief in the value of hip protectors (affective commitment), (ii) acceptance of the clinical efficacy of hip protectors (cognitive commitment), and (iii) a willingness to act or modify their behaviour to generally support the use of hip protectors (behavioural commitment).

## Methods

### Aim, design, and setting

Our aim was to develop and test the psychometric properties of a tool to measure commitment to hip protectors among paid care providers in LTC, named the Commitment to Hip Protectors (C-HiP) Index. To achieve this, we conducted a cross-sectional survey within thirteen non-profit, publically subsidized LTC homes situated in Metro Vancouver and the Fraser Valley, of British Columbia (BC), Canada. Homes ranged from 50 to 234 beds, and all were owned and operated by the Fraser Health Authority.

### Context: Hip protector policy in Fraser health

Fraser Health does not provide hip protectors free of charge to residents living in owned and operated LTC homes, nor are hip protectors reimbursed through national health care coverage (e.g., Medical Services Plan). A single pair of hip protectors costs between $70–$120 CAD.

In 2013, Fraser Health released a clinical practice guideline (CPG) endorsing the use of hip protectors among residents of LTC: (1) with more than 2 falls in the previous 6 months, (2) who were admitted to the home in the past month, (3) with impaired mobility, balance or gait, and (4) who are agitated, restless, or unable to follow instructions. The CPG states that education about hip protectors should be provided to residents who meet this criterion, and if applicable, their family, and any refusal to adhere should be clearly documented in health records.

### Sample

We recruited a convenience sample of 541 paid care providers from publically subsidized LTC homes, who reported working for at least one full month on their floor/neighbourhood/unit, and for at least 8 h per week. We excluded carers who indicated they were unaware of hip protectors (*n* = 5, 0.9%), and one respondent who left this question blank and did not answer any items in the C-HiP Index. An additional six (1.1%) respondents were excluded because they indicated they worked most of their time at privately owned or contracted LTC homes. Another six (1.1%) respondents indicated they worked most of their time at a LTC home owned and operated by the Fraser Health Authority that did not participate in data collection, but as they met our criteria for inclusion, they were included anyway. Of the remaining 529 respondents, the majority were female (90%) and most were health care assistants (55%). About half were full-time (53%), one-quarter were part-time (28%), and the remainder were casual (16%) or unknown. More respondent characteristics are provided in Table 1.

**Table 1:** Demographic characteristics of 529 paid caregivers who completed the C-HiP Index

Characteristics	No. (%)
Gender	
Female	474 (89.6)
Male	40 (7.6)
Missing/unknown	15 (2.8)
Age	
20–29 years	42 (7.9)
30–39 years	87 (16.4)
40–49 years	149 (28.2)
50–59 years	187 (35.3)
60–69 years	46 (8.7)
Missing/unknown	18 (3.4)
Highest level of education	
Less than high school	5 (0.9)
High school or equivalent	43 (8.1)
College or professional certification	312 (59.0)
Bachelor’s degree	119 (22.5)
Master’s degree	36 (6.8)
Missing/unknown	14 (2.6)
Race/ethnicity – mark all that apply	
Black Canadian	
Yes	17 (3.2)
Missing/unknown	24 (4.5)
Caucasian	
Yes	261 (49.3)
Missing/unknown	24 (4.5)
Chinese	
Yes	23 (4.3)
Missing/unknown	24 (4.5)
Filipino	
Yes	69 (13.0)
Missing/unknown	25 (4.7)
South Asian (E.g., East Indian, Pakistani, Sri Lankan)	
Yes	95 (18.0)
Missing/unknown	24 (4.5)
Role/occupation – mark all that apply	
Health care assistant/resident care aide	
Yes	290 (54.8)
Missing/unknown	17 (3.2)
Licensed practical nurse	
Yes	84 (15.9)
Missing/unknown	17 (3.2)
Registered nurse	
Yes	40 (7.6)
Missing/unknown	17 (3.2)
Resident care coordinator	
Yes	13 (2.4)
Missing/unknown	17 (3.2)
Manager	
Yes	14 (2.6)
Missing/unknown	17 (3.2)
Recreational/occupational/physiotherapist	
Yes	24 (4.5)
Missing/unknown	17 (3.2)
Unit/program clerk	
Yes	18 (3.4)
Missing/unknown	17 (3.2)
Employment status	
Part-time	149 (28.2)
Casual	86 (16.2)
Full-time	282 (53.3)
Missing/unknown	12 (2.3)
Shift	
Day shifts	237 (44.8)
Evening shifts	61 (11.5)
Night shifts	11 (2.1)
Combination	206 (38.9)
Missing/unknown	14 (2.6)
Clinical experience	
Less than 1 year	15 (3.0)
1–5 years	98 (18.5)
5–10 years	121 (22.9)
10–20 years	148 (28.0)
20–30 years	94 (17.8)
30 or more years	38 (7.2)
Missing/unknown	15 (2.8)
Organizational tenure	
Less than 1 year	36 (6.8)
1–5 years	159 (30.1)
5–10 years	120 (22.7)
10–20 years	129 (24.4)
20–30 years	57 (10.8)
30 or more years	13 (2.5)
Missing/unknown	15 (2.8)

### C-HiP index development and scoring

Four items were written to measure affective commitment to hip protectors, modified from the affective commitment subscale of Herscovitch and Meyer’s [53] Commitment to Change scale. An example being, “I believe in the value of hip protectors.” Seven items were written to measure behavioural commitment to hip protectors, modified from Mowday, Steers and Porter’s [49] Organizational Commitment Questionnaire (OCQ) and the Compliance and Cooperation subscales of Herscovitch and Meyer’s [53] Measures of Behavioural Support for Change questionnaire. An example being, “I am willing to put in a great deal of effort, above and beyond what is normally expected, to work with hip protectors.” These eleven items were pretested in a convenience sample of 119 paid care providers from two privately owned LTC homes within Fraser Health, and the results were subjected to exploratory factor analysis and internal reliability testing. Two behavioural items were removed due to low pattern matrix and structure matrix coefficients. After pre-testing, six items were added based on qualitative feedback from respondents, constituting the cognitive subscale of the C-HiP Index, an example being, “I am convinced that, when worn, hip protectors reduce risk for injury from falls.” All items used Likert-type response scales ranging from 1 (strongly disagree) to 5 (strongly agree); however, one cognitive item, “I doubt the effectiveness of hip protectors”, used reverse scoring (e.g., a response of 1 is scored as 5-points). Although scores for the C-HiP Index can be calculated either by summing or averaging responses to individual items, we elected to sum responses.

### Protocol

In May 2015, an email message was sent to managers of LTC homes owned and operated by Fraser Health, alerting them of the upcoming study and inviting them to participate. In homes where managers expressed interest in participating, a member of the research team (AMBK) scheduled the launch of data collection.

We developed five different versions of the paper survey. In each, we kept the location of the C-HiP Index the same, but we randomized the order in which individual items were presented within the scale. We also randomized the order in which LTC homes were assigned versions of the paper survey. However, each version was assigned to at least two LTC homes, and all participants from a given LTC home received the same version (i.e., stratified randomization). This method of randomization ensured we received an adequate and relatively equal number of responses to each version of the survey.

During data collection, AMBK offered multiple information sessions within each home to explain the overall aims and objectives of the study and to distribute invitation letters and paper surveys to eligible participants. Additional copies of the invitation letter and paper survey were left behind for those unable to attend sessions (e.g., night shift employees). Once completed, respondents were asked to place paper surveys in a sealed envelope and to leave them in a secured collection box. The return of completed paper surveys was interpreted as implied consent. Data collection lasted between 9 and 10 days in each participating LTC home, and took place between June and December 2015. The Fraser Health Authority Research Ethics Board and the Simon Fraser University Office of Research Ethics approved the study protocol.

### Double data entry

Thirteen volunteers entered the data from returned paper surveys into spreadsheets, including one physiotherapist, five undergraduate students, five graduate students, and two postdoctoral fellows. Paper surveys underwent first and second keying, respectively, each by different volunteers. To facilitate high quality data entry, volunteers were provided with a protocol, adapted from the WHO STEPS Surveillance Manual [60], outlining general rules and guidelines for data entry, including how and when to assign missing data codes and resolutions to common difficulties (e.g., surplus data). All difficulties, and their associated resolutions, were logged on data tracking forms.

The overall error rate was low (1.02%). However, 369 (83.7%) surveys had at least one discrepancy between first and second keying, 197 (44.7%) had at least two errors, and 100 (22.7%) had three or more errors. The data entry supervisor (AMBK) resolved discrepancies by comparing entries to original responses.

### Statistical Methods

Unless otherwise stated, statistical testing was performed using IBM SPSS Statistics version 22.0 (SPSS Inc., Chicago, IL, USA) and significance was defined at the level *p* < 0.05.

#### Missing data

Table 2 describes the amount (count, %) of missing data for each item of the C-HiP Index. 484 (91.5%) respondents answered the C-HiP Index completely. The behavioural item, “When it comes to hip protectors, I am willing to accept changes in the roles and responsibilities of my job”, had the most missing data, with 14 (2.6%) respondents leaving this question blank, while the cognitive item, “I am convinced that, when worn, hip protectors reduce risk for injury from falls”, had the least missing data, with no respondents refusing to answer.

**Table 2:** Missingness, means (SD) and content validity index scores for C-HiP Index items

Item		Missingness		Mean^a^	SD	Content validity index	
		No.	%			Clarity	Relevance
*Affective commitment subscale*							
AFF01	I believe in the value of hip protectors.	2	0.4	4.18	0.88	0.91	1.00
AFF02	Hip protectors are necessary.	6	1.1	4.11	0.93	1.00	0.91
AFF03	Hip protectors are needed.	10	1.9	4.16	0.92	0.91	1.00
AFF04	Hip protectors serve an important purpose.	2	0.4	4.20	0.89	1.00	1.00
*Cognitive commitment subscale*							
COG01	I believe in the effectiveness of hip protectors.	3	0.6	4.16	0.91	0.91	1.00
COG02	I am convinced that, when worn, hip protectors help to protect my residents from injury.	1	0.2	4.17	0.92	1.00	1.00
COG03	I think that hip protectors work.	2	0.4	4.12	0.91	1.00	1.00
COG04	I think that hip protectors are useful.	3	0.6	4.16	0.87	0.91	1.00
COG05	I am convinced that, when worn, hip protectors reduce risk for injury from falls.	0	0	4.17	0.98	1.00	1.00
COG06	I doubt the effectiveness of hip protectors.	7	1.3	2.29	1.19	0.36	0.36
*Behavioural commitment subscale*							
BEH01	I am always willing to work with hip protectors.	5	0.9	4.28	0.77	0.91	1.00
BEH02	I try to remain positive about hip protectors, even under challenging circumstances.	1	0.2	4.21	0.74	0.91	0.91
BEH03	When it comes to hip protectors, I am willing to accept changes in the roles and responsibilities of my job.	14	2.6	4.11	0.82	0.55	0.73
BEH04	I am willing to put in a great deal of effort, above and beyond what is normally expected, to work with hip protectors.	5	0.9	3.98	0.84	0.73	0.82
BEH05	I am willing to adjust the way I do my job, as required to use hip protectors.	4	0.8	4.13	0.80	0.91	0.91

^a^Expectation-Maximization (EM) imputed means; responses ranged from 1 (strongly disagree) to 5 (strongly agree)

We used a combination of single and multiple imputation (MI) procedures to handle missing data. MI is the gold standard of missing data procedures, and is preferred over many commonly used approaches, such as listwise deletion, for its ability to produce unbiased parameter estimates, reasonable estimates of uncertainty (i.e., standard errors and confidence intervals), and maximal statistical power [61, 62]; however, when preparing datasets for preliminary statistical analyses that do not involve standard errors, such as exploratory factor analysis (EFA) and coefficient alpha analyses, the Maximum-Likelihood, single imputation technique known as the Expectation-Maximization (EM) algorithm can be just as useful as MI procedures [62, 63].

Accordingly, we first implemented the EM algorithm to generate a single imputed dataset, from which we: (i) derived means (SD) of individual items within the C-HiP Index; (ii) conducted EFA and alpha coefficient analyses, described in the following sections entitled ‘construct validity’ and ‘internal consistency’, respectively. The EM algorithm contained age, sex, all fifteen items belonging to the C-HiP Index, and single items probing their familiarity with hip protectors, their familiarity with protocols concerning hip protectors at their LTC home, whether they identify as a champion of hip protectors, and whether there is at least one other person in their LTC home that is a champion of hip protectors.

We then used the MI procedures implemented in the mice package [64] of R [65] to prepare for statistical analyses involving hypothesis testing, such as simple linear regression and Mann-Whitney U tests, described in the following sections entitled ‘convergent validity’ and ‘concurrent validity’, respectively. We used a multilevel approach and included all variables from our statistical analyses, along with age, sex and facility code as the Level 2 identifier. We imputed *m* = 5 different datasets. Each statistical test (e.g., simple linear regression) was repeated on all five datasets, and the results from each dataset were pooled to generate a single population estimate, confidence interval, and *p*-value. We pooled population estimates by averaging across the five imputed datasets. We pooled standard errors using the method proposed by Enders (2010), which considers within and between imputed dataset variation [61]. There is currently no established method to pool *p*-values generated from non-parametric tests (i.e., Mann-Whitney U tests) on multiply imputed datasets; however, all *p*-values were *p* < 0.01.

#### Construct validity

Because we conceptualized commitment to hip protectors as subsuming affective, cognitive and behavioural components, with each component subsuming several individual items of the C-HiP Index, we elected to perform hierarchical (i.e., higher order) factor analysis.

Individual C-HiP Index items were first subjected to a series of exploratory factor analysis (EFA) with oblique rotation (i.e., Oblimin with Kaiser Normalization) to identify lower-order factors (i.e., components of commitment). To determine the number of factors to retain in each EFA, we conducted Velicier’s minimum average partial (MAP) tests and parallel analyses [66]. Items were only retained if they had coefficients of .6 or higher on either the pattern matrix or the structure matrix, and they loaded onto the same factor in both matrices. If items did not meet these criteria, they were removed, and a subsequent EFA was performed. This procedure was repeated until all items met the criterion for retention. We then subjected lower-order factors to EFA to identify higher order factors.

For each EFA performed, we report eigenvalues (ranging from 0 to the number of items), which represent the variance in the original data matrix that is reproduced by each of the factors, the percentage of variance explained by each of the factors, and where applicable, factor matrix coefficients, pattern matrix coefficients and/or structure matrix coefficients. We hypothesized hierarchical factor analysis would yield three lower-order factors, representing the three components of commitment, and a single higher-order factor, representing the overarching concept of commitment to hip protectors.

#### Content validity

To assess content validity, a completely anonymous sample of eleven experts, consisting of directors of care and members of the Fraser Health Authority Patient Safety and Injury Prevention Program, rated the relevance and clarity of C-HiP items on Likert scales ranging from 1 (e.g., “Not at all relevant”) to 4 (e.g., “Extremely relevant”). For each item, we computed a content validity index (CVI), taken as the percentage of experts giving a rating of 3 or 4, for both clarity and relevance (e.g., [67]). We hypothesized that each item in the C-HiP Index would have a CVI greater than 0.79 (>79% agreement) for both relevance and clarity, which has been recommended as a threshold of adequate content validity [67]. However, in line with extant research [67], only those items with a CVI less than 0.70 (<70% agreement) for both relevance and clarity were considered unacceptable, and were deleted from the C-HiP Index.

#### Convergent validity

Another method to test the validity of scales is to determine whether variables that ought to be related to the outcome measure of interest are indeed related. An individual’s commitment to hip protectors should ultimately affect how they behave (e.g., [59]). The stronger their commitment, the more likely they should be to engage in a form of discretionary behaviour known as championing (e.g., [53]). A champion is defined as an employee who exhibits considerable personal sacrifice, and goes above and beyond what is explicitly required to serve and enhance the interests of one or more targets within and outside their organization [53]. In general, champions are known for their achievement, persuasiveness, persistence, innovativeness, charisma, enthusiasm, assertiveness, and/or risk-tolerance [68].

Thus, we hypothesized C-HiP Index scores are positively associated with championing. To test this hypothesis, we performed simple linear regression to examine the association between scores on the entire C-HiP Index and responses to the single question, “Do you think of yourself as a champion of hip protectors”, with responses ranging from 1 (strongly disagree) to 5 (strongly agree). We also examined the association between each lower order factor, taken as the sum of items loading onto each factor (e.g., Factor 1) extracted from EFA, and responses to the single question, “Do you think of yourself as a champion of hip protectors”. To minimize interpretation bias, respondents were provided with a definition of championing before being asked to answer.

#### Concurrent validity

To test concurrent validity, we examined the ability of the two lower-order factors and the entire C-HiP Index to distinguish between subgroups of respondents that it should theoretically be able to distinguish between. Although hip protectors substantially reduce risk of hip fracture if worn during a fall, they cannot prevent hip fracture on every occasion, including cases of spontaneous fracture without any obvious external impact (e.g., [69]), when the hip breaks from impact to the buttocks during of a backwards landed fall or a fall to the knees (e.g., [22, 70]), or when the hip protector is not positioned correctly over the greater trochanter (e.g., [71, 72]). Forsen et al. [73] reported that it became increasingly difficult to convince residents of the benefits of hip protectors after each time a hip fracture occurred while wearing a hip protector. Furthermore, an important facilitator of adherence in LTC is the existence of a leader within the home to act as a champion of hip protectors and to convince others of their efficacy [74, 75].

Therefore, we hypothesized that responses to the C-HiP Index should be: (i) lower among paid caregivers who responded ‘Yes’ to the question, “Are you aware of a resident breaking their hip during a fall while wearing a hip protector”, compared to those who responded ‘No’; (ii) higher among paid caregivers who agreed (responded ‘4’ or ‘5’ on a 5-point Likert scale) with the statement, “Would you say there is at least one other person in your residential care facility that is a champion of hip protectors”, compared to those who did not agree (responded ‘1’, ‘2’ or ‘3’). As C-HiP Index responses were rightward skewed, we performed Mann-Whitney U tests to determine whether there were differences in median responses to lower-order factors and the entire C-HiP Index between these subgroups of respondents.

#### Internal consistency

To assess internal consistency, we computed Cronbach’s alpha coefficients for lower-order factors and the entire C-HiP Index. We hypothesized alpha coefficients would be above 0.70, indicating acceptable internal consistency [76].

## Results

Table 2 describes mean (SD) responses to individual items of the C-HiP Index derived from the EM imputed dataset.

### Construct validity

Contrary to expectation, both Velicier’s MAP test and parallel analysis indicated the presence of only two lower-order factors. The eigenvalues of Factor 1 and Factor 2 were 9.225 and 1.078, respectively. Accordingly, lower-order factors explained 68.7% of the variance in responses. One cognitive item (i.e., COG06) – “I doubt the effectiveness of hip protectors” – had poor pattern matrix and structure matrix coefficients for both Factor 1 (−.597, −.508, respectively) and Factor 2 (.136, −.250, respectively), and was removed. After removal of COG06, EFA yielded two lower-order factors, with eigenvalues of 9.002 and 1.033, respectively. Lower-order factors now explained 71.7% of the variance in responses. Items from the affective and cognitive subscales loaded highest on Factor 1, whereas items from the behavioural subscale loaded highest on Factor 2 (Table 3).

**Table 3:** Pattern and structure matrix coefficients for each item retained in exploratory factor analysis

Item		Pattern matrix		Structure matrix	
		Factor 1	Factor 2	Factor 1	Factor 2
*Affective commitment subscale*					
AFF01	I believe in the value of hip protectors.	.861	.058	.902	.656
AFF02	Hip protectors are necessary.	.847	.015	.857	.602
AFF03	Hip protectors are needed.	.825	.040	.853	.612
AFF04	Hip protectors serve an important purpose.	.853	.063	.897	.655
*Cognitive commitment subscale*					
COG01	I believe in the effectiveness of hip protectors.	.933	−.025	.915	.622
COG02	I am convinced that, when worn, hip protectors help to protect my residents from injury.	.908	−.035	.884	.595
COG03	I think that hip protectors work.	.926	−.019	.913	.624
COG04	I think that hip protectors are useful.	.898	.027	.917	.650
COG05	I am convinced that, when worn, hip protectors reduce risk for injury from falls.	.891	−.030	.870	.588
*Behavioural commitment subscale*					
BEH01	I am always willing to work with hip protectors.	.202	.620	.633	.761
BEH02	I try to remain positive about hip protectors, even under challenging circumstances.	.248	.488	.587	.660
BEH03	When it comes to hip protectors, I am willing to accept changes in the roles and responsibilities of my job.	−.070	.760	.457	.711
BEH04	I am willing to put in a great deal of effort, above and beyond what is normally expected, to work with hip protectors.	.137	.653	.589	.747
BEH05	I am willing to adjust the way I do my job, as required to use hip protectors.	−.100	.935	.549	.866

**Fig. 1:**
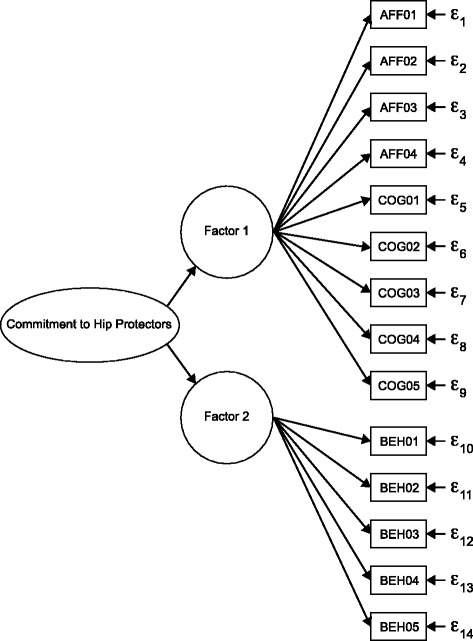
Conceptual model of commitment to hip protectors determined using hierarchical (higher-order) factor analysis

Higher-order factor analysis supported a hierarchical factor structure (Fig. 1). Both Factor 1 (affective/cognitive subscale) and Factor 2 (behavioural subscale) loaded onto a single higher-order factor, “commitment to hip protectors,” having an eigenvalue of 1.386 and accounting for 69.3% of the variance in responses. Factor 1 (affective/cognitive subscale) and Factor 2 (behavioural subscale) each had factor matrix coefficients of .833.

### Content validity

Twelve items had a CVI above 0.79 for both clarity and relevance. A single item, BEH04 (“I am willing to put in a great deal of effort, above and beyond what is normally expected, to work with hip protectors”), had a CVI of 0.82 (‘adequate’) for relevance, but a CVI of 0.73 (‘questionable’) for clarity. Another item, BEH03 (“When it comes to hip protectors, I am willing to accept changes in the roles and responsibilities of my job”), had a CVI of 0.73 (‘questionable’) for relevance, and a CVI of 0.55 (‘unacceptable’) for clarity. Finally, a single item, COG06 (“I doubt the effectiveness of hip protectors”) had a CVI below 0.70 for both clarity and relevance, and therefore, was eliminated from the C-HiP Index (Table 2).

### Convergent validity

After removal of COG06, a 1-unit increase in championing (responses to the single question, “Do you think of yourself as a champion of hip protectors”, scored from 1 to 5) was associated with 3.6-point (95% CI: 2.9–4.2; *p* < 0.01), 1.6-point (95% CI: 1.4–1.9; *p* < 0.01) and 5.2-point (95% CI: 4.4–6.1; *p* < 0.01) increases in the affective/cognitive subscale (scored from 9 to 45), the behavioural subscale (scored from 5 to 25) and the entire C-HiP Index (scored from 14 to 70), respectively.

### Concurrent validity

We observed significantly lower median responses to the affective/cognitive subscale (estimated difference = 4.0-points; *p* < 0.01), the behavioural subscale (estimated difference = 1.0-point; *p* < 0.01), and the entire C-HiP Index (estimated difference = 4.3-points; *p* < 0.01) among paid care providers who were aware of a resident breaking their hip during a fall while wearing a hip protector compared to those who were unaware (Table 4).

**Table 4:** Concurrent validity of the C-HiP Index

Characteristic	Affective/Cognitive^a^		Behavioural		C-HiP index^a^	
	Median	*P*-value	Median	*P*-value	Median	*P*-value
Aware of padded hip fracture						
Yes (*n* = 203, 38%)	36.0	*p* < 0.01*	20.0	*p* < 0.01*	56.0	*p* < 0.01*
No (*n* = 326, 62%)	40.0		21.0		60.3	
Existence of a champion						
Yes (*n* = 397, 75%)	40.0	*p* < 0.01*	21.0	*p* < 0.01*	61.0	*p* < 0.01*
No (*n* = 132, 25%)	35.0		19.0		54.0	

* *p* < 0.01 in each multiply imputed dataset; Mann-Whitney U tests

^a^After removal of the cognitive item (COG06), “I doubt the effectiveness of hip protectors”

We also observed significantly higher median responses to the affective/cognitive subscale (estimated difference = 5.0-points; *p* < 0.01), the behavioural subscale (estimated difference = 2.0-points; *p* < 0.01), and the entire C-HiP Index (estimated difference = 7.0-points; *p* < 0.01) among paid care providers who agreed that there is at least one other person in their LTC home that is a champion of hip protectors compared to those who did not agree (Table 4).

### Internal consistency

After removal of COG06, Cronbach’s alpha coefficients for the affective/cognitive subscale, the behavioural subscale, and the entire C-HiP Index were 0.97, 0.87, and 0.96, respectively.

## Discussion

Hip protectors represent a promising technology for the prevention of hip fractures in one of society’s frailest and most cognitively impaired cohorts of older adults, residents of LTC. However, a major barrier to the clinical effectiveness of hip protectors in LTC is poor user adherence in the wearing of hip protectors, often dropping below 50% in clinical trials [36]. Within LTC, care providers are believed to play a particularly important role in influencing a resident’s decision to wear hip protectors on a regular basis (e.g., [39–46]). The overall commitment of paid care providers towards hip protectors has been identified as an important determinant of adherence, but empirical evidence is lacking [37]. To address this knowledge gap, our aim was to develop a valid and reliable tool to measure commitment to hip protectors among paid caregivers in LTC.

Consistent with extant research, we defined commitment to hip protectors as an individual’s attachment to and behavioural intentions towards hip protectors, reflected by three components: (i) a belief in the value and importance of hip protectors (affective commitment), (ii) acceptance of the clinical efficacy of hip protectors (cognitive commitment), and (iii) a willingness to act or modify their behaviour to generally support the use of hip protectors (behavioural commitment). We adapted existing metrics of workplace commitment to develop the C-HiP Index, originally containing the 4-item affective subscale, the 6-item cognitive subscale, and the 5-item behavioural subscale. However, one negative item was removed from the cognitive subscale, as it did not meet our criteria for retention in EFA and it had a CVI less than 0.70 (<70% agreement) for both clarity and relevance. Despite expert ratings of unacceptable and questionable clarity, respectively, we retained BEH03 and BEH04 in their original form as they met our criteria for retention in EFA and did not have unacceptably low CVI scores (<0.70) for relevance.

We expected EFA to confirm a hierarchical factor structure, yielding three lower-order factors, and a single higher-order factor. We hypothesized items from the affective subscale would load onto a first lower-order factor, items from the cognitive subscale would load onto a second lower-order factor, and items from the behavioural subscale would load onto a third lower-order factor. We also hypothesized lower-order factors would subsequently load onto a single higher-order factor. However, contrary to expectation, EFA supported a hierarchical factor structure with only two lower-order factors, and a single higher-order factor. Items from the affective and cognitive subscales loaded together onto a first lower-order factor (i.e., ‘Factor 1’) and those from the behavioural subscale loaded onto a second lower-order factor (i.e., ‘Factor 2’). Both lower-order factors then loaded onto a single higher-order factor (i.e., ‘Commitment to Hip Protectors’).

We have shown it is hard to separate affective commitment from cognitive commitment, and believe this inconsistency is explained by empirical, rather than conceptual problems. Firstly, it is possible that these results are merely a by-product of our sampling strategy, in which we recruited participants from a single health authority in BC, Canada. Although excessive variability (i.e., noise) in any signal can interfere with our ability to make statistical inferences, the same can be said when there is too little variability. For example, we need variability in predictor variables to explain variability in outcomes. After conducting this study, it became apparent that commitment to hip protectors among paid caregivers in participating LTC homes was higher and less variable than expected, with mean scores exceeding 4.00 for most (93%) items of the C-HiP Index. Therefore, we may not have been able to distinguish affective from cognitive commitment because the majority of respondents believed in the value of hip protectors and their clinical efficacy. To determine if affective commitment and cognitive commitment truly are inseparable, future research should include participants from LTC homes where hip protectors have not been embraced as a fall-related injury prevention strategy, and commitment to hip protectors is much lower.

Alternatively, we might have failed to capture the true essence of affective commitment to hip protectors in the C-HiP Index, and instead wrote 9-items to measure cognitive commitment. Solinger, van Olffen and Roe [57] defined affective commitment as an emotional attachment and identification with one or more targets, and cognitive commitment as the internalization of a target’s goals, norms and values. Although we adapted affective items from the well-validated affective commitment subscale of Herscovitch and Meyer’s [53] Commitment to Change scale, in future research it might be fruitful to revise and improve this subscale of the C-HiP Index to better capture an individual’s emotional attachment to and identification with hip protectors. Examples could include, “I love the idea of hip protectors”, “I hate the idea of hip protectors”, “I would consider wearing hip protectors if I was a senior residing in this long-term care home”, or perhaps, “I would put hip protectors on someone I love or care deeply for”.

Despite the unexpected factor structure, we were able to demonstrate the content, convergent and concurrent validity of the C-HiP Index. For example, after removing COG06, 86% (12 of 14) of remaining C-HiP Index items had a CVI above 0.79 for both clarity and relevance. Furthermore, we saw a positive and significant association of overall C-HiP Index scores with self-reported championing behaviours (estimated slope of the regression line, β_1_ = 5.2-points). As theorized, respondents who were aware of a resident breaking their hip during a fall while wearing a hip protector had lower median scores compared to those who were unaware (estimated difference = 4.3-points). Also, respondents who agreed with the statement that there is at least one other person in their LTC home that is a champion of hip protectors had higher median scores compared to those who did not agree (estimated difference = 7.0-points). These findings are in line with those from previous qualitative and quantitative studies examining resident and staff experiences using hip protectors, which suggest differences should exist in commitment between these subgroups of respondents [73–75].

In general, the C-HiP Index demonstrated acceptable internal consistency, having alpha values greater than 0.70 [76]. Alpha values of the affective/cognitive subscale (9-items), the behavioural subscale (5-items), and the entire C-HiP Index (14-items) were 0.97, 0.87, and 0.96, respectively, thereby achieving high consistency, but also suggesting that some items could potentially be removed from the affective/cognitive subscale to reduce redundancies [77]. However, as Cronbach’s alpha coefficients are sensitive to the number of the questions contained in a scale, with longer scales always demonstrating improved reliability, these findings are not surprising, and thus, should be interpreted with some caution.

We acknowledge some important limitations. First, we recruited a convenience sample of paid care providers, and did not adopt a random sampling strategy. Thus, it is possible that those who chose to participate in our study are more committed to hip protectors than those who declined, which could have introduced bias. However, we are fairly confident our sampling strategy has not favoured and/or excluded obvious groups within the target population in terms of gender, age, employment status, and occupation/role type, as distributions are consistent with those reported in previous studies (e.g., [78]), including the National Study of Long-Term Care Providers conducted by the National Center for Health Statistics in the United States [79]. Second, previous research has shown that attitudes towards hip protectors differ between caregivers working day and night shifts, with night shift employees reporting less favourable attitudes towards hip protectors [47]. For example, Milisen et al. [47] observed that nurses working night shifts were more likely to rate a hip protector policy in LTC as time-consuming, stressful, and as having a potentially negative impact on the independence of residents compared to nurses working day shifts only. It is possible that care providers who work mostly nightshifts might respond to the C-HiP Index in a conceptually distinct manner to those working day or evening shifts. As we received only *n* = 11 responses from night shift employees, we could not examine whether the factor structure of C-HiP Index items was equivalent between day/evening and night shift respondents. Third, we only recruited participants from a single health authority, which limits the generalizability of our findings outside the Fraser Valley of BC, Canada. Regions located beyond these borders might have differing policies on hip protectors, cultural norms, educational requirements, and use of dialects and/or languages, which could affect responses to the C-HiP Index and consequently, measures of validity and reliability obtained from psychometric testing. Fourth, although we conducted a comprehensive evaluation of the C-HiP Index, we did not explore face validity. Face validity can be assessed by asking end-users to subjectively rate the clarity/transparency and relevance of the instrument as it appears to them at face value. Finally, when assessing content validity, we did not collect data on the first language of respondents, which might have provided valuable insight into why experts rated the clarity of BEH03, BEH04 and COG06 as questionable (CVI = 0.70–0.79) or unacceptable (CVI < 0.70). An understanding of how clarity rankings may have associated with first language could aide future endeavours to improve the C-HiP Index.

## Conclusions

Despite these limitations, we offer novel insight into the psychometric properties of a tool to measure commitment to hip protectors among paid care providers in LTC. We have provided evidence of the content, construct, convergent, and concurrent validity, as well as the internal consistency of the C-HiP Index. The development of a valid and reliable assessment tool is a crucial first step in understanding the relationship between care provider commitment and levels of reported adherence in the wearing of hip protectors amongst residents of LTC. Downstream, findings have the potential to improve the safety and efficiency of care for institutionalized older adults, through deeper understanding of the factors governing adherence to a promising technology for the prevention of fall-related hip fractures, wearable hip protectors.

## Abbreviations

LTC: Long-Term Care
BC: British Columbia
CPG: Clinical Practice Guideline
MI: Multiple Imputation
EM: Expectation-Maximization
SD: Standard Deviation
EFA: Exploratory Factor Analysis
CVI: Content Validity Index
ICC: Intraclass Correlation

## References

[1] Kramarow E, Chen LH, Hedegaard H, Warner M. Deaths from unintentional injury among adults aged 65 and over: United States, 2000–2013. NCHS data brief, no 199. Hyattsville, MD: National Center for Health Statistics. 2015. 25973998

[2] Public Health Agency of Canada. Seniors' falls in Canada: second report. Ottawa, ON: Public Health Agency of Canada = Agence de la santé publique du Canada; 2014.

[3] Scott VJ, Elliott S, Wagar L, Public Health Agency of Canada. Division of aging and seniors., Canadian electronic library (firm). Falls & related injuries among older Canadians: fall-related hospitalizations & prevention initiatives. Ottawa, Ont.: Public Health Agency of Canada; 2010.

[4] Parachute. The cost of injury in Canada. Toronto, Ont.: Parachute; 2015.

[5] Berry SD, Samelson EJ, Bordes M, Broe K, Kiel DP (2009). Survival of aged nursing home residents with hip fracture. J Gerontol A Biol Sci Med Sci.

[6] Fransen M, Woodward M, Norton R, Robinson E, Butler M, Campbell AJ (2002). Excess mortality or institutionalization after hip fracture: men are at greater risk than women. J Am Geriatr Soc.

[7] Neuman MD, Silber JH, Magaziner JS, Passarella MA, Mehta S, Werner RM. Survival and functional outcomes after hip fracture among nursing home residents. JAMA Intern Med. 2014. doi:10.1001/jamainternmed.2014.2362. 10.1001/jamainternmed.2014.2362 PMC4122620 25055155

[8] Slor CJ, Adamis D, Jansen RW, Meagher DJ, Witlox J, Houdijk AP (2013). Delirium motor subtypes in elderly hip fracture patients: risk factors, outcomes and longitudinal stability. J Psychosom Res.

[9] Slor CJ, Witlox J, Jansen RW, Adamis D, Meagher DJ, Tieken E (2013). Affective functioning after delirium in elderly hip fracture patients. Int Psychogeriatr.

[10] Visschedijk J, Achterberg W, Van Balen R, Hertogh C (2010). Fear of falling after hip fracture: a systematic review of measurement instruments, prevalence, interventions, and related factors. J Am Geriatr Soc.

[11] Williams LJ, Berk M, Henry MJ, Stuart AL, Brennan SL, Jacka FN (2014). Depression following fracture in women: a study of age-matched cohorts. BMJ Open.

[12] Kanwar A, Singh M, Lennon R, Ghanta K, McNallan SM, Roger VL (2013). Frailty and health-related quality of life among residents of long-term care facilities. J Aging Health.

[13] Fried TR, Mor V (1997). Frailty and hospitalization of long-term stay nursing home residents. J Am Geriatr Soc.

[14] Butler M, Norton R, Lee-Joe T, Cheng A, Campbell AJ (1996). The risks of hip fracture in older people from private homes and institutions. Age Ageing.

[15] Norton R, Campbell AJ, Reid IR, Butler M, Currie R, Robinson E (1999). Residential status and risk of hip fracture. Age Ageing.

[16] Ooms ME, Vlasman P, Lips P, Nauta J, Bouter LM, Valkenburg HA (1994). The incidence of hip fractures in independent and institutionalized elderly people. Osteoporos Int.

[17] Nikitovic M, Wodchis WP, Krahn MD, Cadarette SM (2013). Direct health-care costs attributed to hip fractures among seniors: a matched cohort study. Osteoporos Int.

[18] Cummings SR, Nevitt MC (1994). Non-skeletal determinants of fractures: the potential importance of the mechanics of falls. Study of osteoporotic fractures research group. Osteoporos Int.

[19] Greenspan SL, Myers ER, Maitland LA, Resnick NM, Hayes WC (1994). Fall severity and bone mineral density as risk factors for hip fracture in ambulatory elderly. JAMA.

[20] Hayes WC, Myers ER, Morris JN, Gerhart TN, Yett HS, Lipsitz LA (1993). Impact near the hip dominates fracture risk in elderly nursing home residents who fall. Calcif Tissue Int.

[21] Nevitt MC, Cummings SR (1993). Type of fall and risk of hip and wrist fractures: the study of osteoporotic fractures. The study of osteoporotic fractures research group. J Am Geriatr Soc.

[22] Yang Y, Mackey DC, Liu-Ambrose T, Feldman F, Robinovitch SN (2016). Risk factors for hip impact during real-life falls captured on video in long-term care. Osteoporos Int.

[23] Colon-Emeric CS, Datta SK, Matchar DB (2003). An economic analysis of external hip protector use in ambulatory nursing facility residents. Age Ageing.

[24] Gandjour A, Weyler EJ (2008). Cost-effectiveness of preventing hip fractures by hip protectors in elderly institutionalized residents in Germany. Value Health.

[25] Honkanen LA, Schackman BR, Mushlin AI, Lachs MS (2005). A cost-benefit analysis of external hip protectors in the nursing home setting. J Am Geriatr Soc.

[26] Sawka AM, Gafni A, Boulos P, Beattie K, Papaioannou A, Cranney A (2007). Could a policy of provision of hip protectors to elderly nursing home residents result in cost savings in acute hip fracture care? The case of Ontario. Canada Osteoporos Int.

[27] Singh S, Sun H, Anis AH (2004). Cost-effectiveness of hip protectors in the prevention of osteoporosis related hip fractures in elderly nursing home residents. J Rheumatol.

[28] Cameron ID, Gillespie LD, Robertson MC, Murray GR, Hill KD, Cumming RG (2012). Interventions for preventing falls in older people in care facilities and hospitals. Cochrane Database Syst Rev.

[29] Laing AC, Robinovitch SN (2008). The force attenuation provided by hip protectors depends on impact velocity, pelvic size, and soft tissue stiffness. J Biomech Eng.

[30] Laing AC, Robinovitch SN (2008). Effect of soft shell hip protectors on pressure distribution to the hip during sideways falls. Osteoporos Int.

[31] Cameron ID, Cumming RG, Kurrle SE, Quine S, Lockwood K, Salkeld G (2003). A randomised trial of hip protector use by frail older women living in their own homes. Inj Prev..

[32] Forsen L, Sogaard AJ, Sandvig S, Schuller A, Roed U, Arstad C (2004). Risk of hip fracture in protected and unprotected falls in nursing homes in Norway. Inj Prev..

[33] Kannus P, Parkkari J, Niemi S, Pasanen M, Palvanen M, Jarvinen M (2000). Prevention of hip fracture in elderly people with use of a hip protector. New Engl J Med.

[34] O'Halloran PD, Cran GW, Beringer TR, Kernohan G, O'Neill C, Orr J (2004). A cluster randomised controlled trial to evaluate a policy of making hip protectors available to residents of nursing homes. Age Ageing.

[35] Koike T, Orito Y, Toyoda H, Tada M, Sugama R, Hoshino M (2009). External hip protectors are effective for the elderly with higher-than-average risk factors for hip fractures. Osteoporos Int.

[36] Santesso N, Carrasco-Labra A, Brignardello-Petersen R (2014). Hip protectors for preventing hip fractures in older people. Cochrane Database Syst Rev.

[37] Korall AM, Feldman F, Scott VJ, Wasdell M, Gillan R, Ross D (2015). Facilitators of and barriers to hip protector acceptance and adherence in long-term care facilities: a systematic review. J Am Med Dir Assoc.

[38] Mitoku K, Masaki N, Ogata Y, Okamoto K (2016). Vision and hearing impairments, cognitive impairment and mortality among long-term care recipients: a population-based cohort study. BMC Geriatr.

[39] Burl JB, Centola J, Bonner A, Burque C (2003). Hip protector compliance: a 13-month study on factors and cost in a long-term care facility. J Am Med Dir Assoc.

[40] Cryer C, Knox A, Martin D, Barlow J (2002). Cantebury hip protector project team. Hip protector compliance among older people living in residential care homes. Inj Prev..

[41] Cryer C, Knox A, Stevenson E (2008). Factors associated with hip protector adherence among older people in residential care. Inj Prev..

[42] Tavener-Smith K, De Vet G (2006). Further exploring hip protector use. Australas J Ageing.

[43] Thompson P, Jones C, Dawson A, Thomas P, Villar T (2005). An in-service evaluation of hip protector use in residential homes. Age Ageing.

[44] Warnke A, Meyer G, Bender R, Muhlhauser I (2004). Predictors of adherence to the use of hip protectors in nursing home residents. J Am Geriatr Soc.

[45] van Schoor NM, Asma G, Smit JH, Bouter LM, Lips P (2003). The Amsterdam hip protector study: compliance and determinants of compliance. Osteoporos Int.

[46] Zimmerman S, Magaziner J, Birge SJ, Barton BA, Kronsberg SS, Kiel DP (2010). Adherence to hip protectors and implications for U.S. long-term care settings. J Am Med Dir Assoc.

[47] Milisen K, Coussement J, Boonen S, Geeraerts A, Druyts L, Van Wesenbeeck A (2011). Nursing staff attitudes of hip protector use in long-term care, and differences in characteristics between adherent and non-adherent residents: a survey and observational study. Int J Nurs Stud.

[48] Meyer JP, Allen NJ (1991). A three-component conceptualization of organizational commitment. Hum Resour Manag R..

[49] Mowday RT, Steers RM, Porter LW (1979). The measurement of organizational commitment. J Vocat Behav.

[50] Clugston M, Howell JP, Dorfman PW (2000). Does cultural socialization predict multiple bases and foci of commitment?. J Manag.

[51] Stinglhamber F, Bentein K, Vandenberghe C (2002). Extension of the three-component model of commitment to five foci: development of measures and substantive test. Eur J Psychol Assess.

[52] Meyer JP, Allen NJ, Smith CA (1993). Commitment to organizations and occupations: Extension and test of a three-component conceptualization. J Appl Psychol..

[53] Herscovitch L, Meyer JP (2002). Commitment to organizational change: Extension of a three-component model. J Appl Psychol..

[54] Hunter GK, Panagopoulos NG (2015). Commitment to technological change, sales force intelligence norms, and salesperson key outcomes. Ind Market Manag.

[55] Meyer JP, Herscovitch L (2001). Commitment in the workplace: toward a general model. Hum Resour Manag R.

[56] Jaros SJ, Jermier JM, Koehler JW, Sincich T (1993). Effects of continuance, affective, and moral commitment on the withdrawal process: an evaluation of eight structural equation models. Acad Manag J.

[57] Solinger ON, van Olffen W, Roe RA (2008). Beyond the three-component model of organizational commitment. J Appl Psychol.

[58] Ajzen I, Fishbein M (1980). Understanding attitudes and predicting social behavior.

[59] Eagly AH, Chaiken S (1993). The psychology of attitudes.

[60] World Health Organization (2005). WHO STEPS surveillance manual: the WHO STEPwise approach to chronic disease risk factor surveillance.

[61] Enders CK (2010). Applied missing data analysis.

[62] Schafer JL, Graham JW (2002). Missing data: our view of the state of the art. Psychol Methods.

[63] Graham JW (2009). Missing data analysis: making it work in the real world. Annu Rev Psychol.

[64] van Buuren S, Groothuis-Ousdhoorn K (2011). Mice: multivariate imputation by chained equations in R. J Stat Softw.

[65] R Core Team. R: a language and environment for statistical computing. Austria, Vienna: R Foundation for Statistical Computing; 2016. Retrieved from http://www.r-project.org/.

[66] O'Connor BP (2000). SPSS and SAS programs for determining the number of components using parallel analysis and Velicer's MAP test. Behav Res Methods Instrum Comput.

[67] Hyrkäs K, Appelqvist-Schmidlechner K, Oksa L (2003). Validating an instrument for clinical supervision using an expert panel. Int J Nurs Stud.

[68] Shea CM, Belden CM (2016). What is the extent of research on the characteristics, behaviors, and impacts of health information technology champions? A scoping review. BMC Med Inform Decis.

[69] Michelson JD, Myers A, Jinnah R, Cox Q, Van Natta M (1995). Epidemiology of hip fractures among the elderly. Risk factors for fracture type. Clin Orthop Rel Res.

[70] Kelly DW, Kelly BD (2012). A novel diagnostic sign of hip fracture mechanism in ground level falls: two case reports and review of the literature. J Med Case Rep.

[71] Choi WJ, Hoffer JA, Robinovitch SN (2010). The effect of positioning on the biomechanical performance of soft shell hip protectors. J Biomech.

[72] Minns RJ, Marsh AM, Chuck A, Todd J (2007). Are hip protectors correctly positioned in use?. Age Ageing.

[73] Forsen L, Sandvig S, Schuller A, Sogaard AJ (2004). Compliance with external hip protectors in nursing homes in Norway. Inj Prev.

[74] Davies S, Doherty D, Glover J, Johnson T (2004). Preventing hip fractures in care homes 2: role of the specialist nurse. Br J Nurs.

[75] Doherty D, Glover J, Davies S, Johnson T (2004). Preventing hip fracture in care homes 1: views of residents and staff. Br J Nurs.

[76] Bland JM, Altman DG (1997). Cronbach's alpha. BMJ.

[77] Tavakol M, Dennick R (2011). Making sense of Cronbach's alpha. Int J Med Educ.

[78] Ngan K, Drebit S, Siow S, Yu S, Keen D, Alamgir H (2010). Risks and causes of musculoskeletal injuries among health care workers. Occup Med.

[79] Harris-Kojetin L, Sengupta M, Park-Lee E, Valverde R, Caffrey C, Rome V et al. Long-term care providers and services users in the United States: data from the National Study of long-term care providers, 2013-2014. Vital health stat 3. 2016(38):x-xii; 1-105. 27023287

